# Tailbeat perturbations improve swimming efficiency by reducing the phase lag between body motion and the resulting fluid response

**DOI:** 10.1093/pnasnexus/pgae073

**Published:** 2024-02-17

**Authors:** Li-Ming Chao, Laibing Jia, Siyuan Wang, Alexander Liberzon, Sridhar Ravi, Iain D Couzin, Liang Li

**Affiliations:** Department of Collective Behaviour, Max Planck Institute of Animal Behavior, Konstanz 78464, Germany; Centre for the Advanced Study of Collective Behaviour, University of Konstanz, Konstanz 78464, Germany; Department of Biology, University of Konstanz, Konstanz 78464, Germany; Department of Naval Architecture, Ocean and Marine Engineering, University of Strathclyde, Glasgow G4 0LZ, UK; Department of Collective Behaviour, Max Planck Institute of Animal Behavior, Konstanz 78464, Germany; Centre for the Advanced Study of Collective Behaviour, University of Konstanz, Konstanz 78464, Germany; Department of Biology, University of Konstanz, Konstanz 78464, Germany; School of Mechanical Engineering, Tel Aviv University, Tel Aviv 69978, Israel; School of Engineering and Information Technology, University of New South Wales, Canberra, ACT 2610, Australia; Department of Collective Behaviour, Max Planck Institute of Animal Behavior, Konstanz 78464, Germany; Centre for the Advanced Study of Collective Behaviour, University of Konstanz, Konstanz 78464, Germany; Department of Biology, University of Konstanz, Konstanz 78464, Germany; Department of Collective Behaviour, Max Planck Institute of Animal Behavior, Konstanz 78464, Germany; Centre for the Advanced Study of Collective Behaviour, University of Konstanz, Konstanz 78464, Germany; Department of Biology, University of Konstanz, Konstanz 78464, Germany

**Keywords:** swimming efficiency, perturbations, hydrodynamics, computational fluid dynamics, robotics

## Abstract

Understanding how animals swim efficiently and generate high thrust in complex fluid environments is of considerable interest to researchers in various fields, including biology, physics, and engineering. However, the influence of often-overlooked perturbations on swimming fish remains largely unexplored. Here, we investigate the propulsion generated by oscillating tailbeats with superimposed rhythmic perturbations of high frequency and low amplitude. We reveal, using a combination of experiments in a biomimetic fish-like robotic platform, computational fluid dynamics simulations, and theoretical analysis, that rhythmic perturbations can significantly increase both swimming efficiency and thrust production. The introduction of perturbations increases pressure-induced thrust, while reduced phase lag between body motion and the subsequent fluid dynamics response improves swimming efficiency. Moreover, our findings suggest that beneficial perturbations are sensitive to kinematic parameters, resolving previous conflicts regarding the effects of such perturbations. Our results highlight the potential benefits of introducing perturbations in propulsion generators, providing potential hypotheses for living systems and inspiring the design of artificial flapping-based propulsion systems.

**Significance Statement**

From birds in the sky to fish beneath the water, organisms exemplify efficient movement through flapping propulsion. Understanding the mechanism behind this high-efficiency propulsion is invaluable across biological studies, fluid mechanics, and engineering. In our study, by superimposing high-frequency, low-amplitude rhythmic perturbations to a basic flapping oscillation, we surprisingly found both thrust and swimming efficiency could be improved in robotic experiments and computational fluid dynamics simulations. Further analyses suggest that superimposing these rhythmic perturbations amplifies the pressure-induced thrust, and reduces phase lag between body motion and fluid dynamics response, thereby boosting swimming efficiency. Our study hypothesizes that biology may employ similar mechanisms to optimize efficiency and provides a mechanism for engineers to design highly efficient vehicles driven by flapping.

## Introduction

Fish have evolved exceptional swimming abilities over almost half a billion years of evolution (1, 2), which has drawn significant interest from various fields such as biology, engineering, physics, and mathematics (3–5). Previous research has shown that fish can enhance their swimming efficiency through a variety of adaptations, including reducing drag with refined surface structures (6–8), harnessing energy from flows created by nearby fish (9–14), adjusting the flexibility of their tail (15–18), and optimizing their swimming movements (19–22).

Being so fundamental, how fish optimize swimming kinematics to enhance swimming efficiency has been widely explored over the past decades (23). A prevailing understanding from biological (24–27) and physical (28, 29) investigations has revealed that swimming in a Strouhal number (defined as St=2fA/U, where *f* is the swimming frequency, *A* denotes tailbeats amplitude, and *U* refers to the swimming velocity) of 0.2–0.4 allows individuals to achieve optimal swimming efficiency. Besides Strouhal number, other studies have shown that swimming efficiency also benefits from other kinematic properties, such as the amplitude of tailbeat (30), the reduced frequency (normalized swimming frequency) (31), and the profile of oscillating waveforms (e.g. nonsinusoidal (32, 33) and quasisymmetric oscillations (34, 35)). Saadat et al. (30) revealed that a Strouhal number of 0.2–0.4 in combination with an amplitude of approximately 20% of the body length can lead to efficient propulsion. Floryan et al. (31) found, within a specific Strouhal number, the smaller the reduced frequency, the higher the propulsion efficiency.

Most current studies on swimming kinematics and performance assume that the swimmer’s movements can be described using various idealized sinusoidal oscillations. However, in nature, swimmers do not use such “clean” kinematic oscillations to move forward. With fast Fourier transform algorithm, natural kinematic oscillations can be resolved into basic and high-frequency components. In most studies, the high-frequency components, which are typically low in amplitude, are often disregarded in simplified body wave models due to their similarity to noise (36). As a result, few studies have investigated the role of these high-frequency and low-amplitude perturbations in kinematics (37, 38). Lehn et al. (37) found that combining high-frequency and low-amplitude perturbations to flexible flapping foils can increase thrust and efficiency. However, it is unclear whether this improvement is mainly due to the flexibility of the foil or the perturbations, as certain stiffness of the foil can also significantly improve efficiency (15–17). Furthermore, some studies that examined the performance of a flapping rigid foil found that perturbations can enhance thrust but not the efficiency of swimming (38), thus calling into question the mechanism that allows for increased efficiency with perturbations. In addition, previous studies have primarily investigated how perturbation frequency impacts swimming efficiency, while there is a lack of research exploring the effect of perturbation amplitude, to thrust production and efficiency, as well as the combined impact of both frequency and amplitude. Overall, it is still largely unknown whether a swimmer with perturbations can improve thrust and efficiency simultaneously, and if so, what kind of perturbations are beneficial and how they lead to improvement. In this work, we combined experimental, numerical, and theoretical studies to systematically explore the benefits of perturbations over the swimmer’s performance (Fig. S1).

## Results

### Robotic fish experiments

**Fig. 1.**
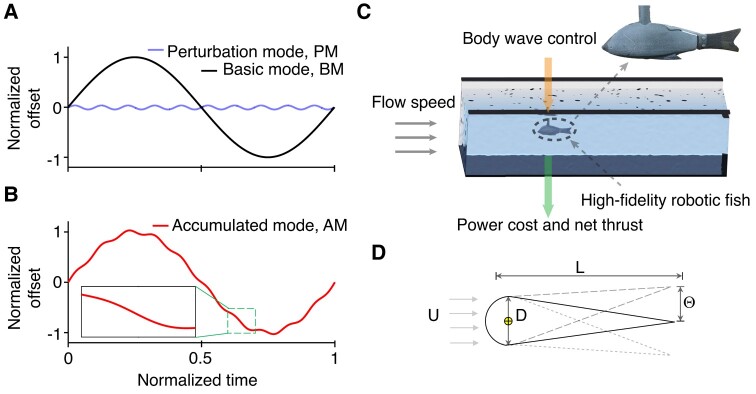
A and B) Foil pitching angle variation in time for the periodic “basic mode” (BM), “perturbation mode” PM (controlled by $\left(\;\;\tilde{f},\tilde{A})=(10,0.04\right)$), and “accumulated mode” AM (controlled by $\left(\;\;\tilde{f},\tilde{A})=(10,0.04\right)$), respectively, where the time is normalized by $1/f_{\mathrm{bm}}$. C) Sketch of the experiments in the flow tank with a high-fidelity robotic fish. The motion of the robotic fish’s body wave was manipulated with and without perturbations (BM and AM), and the power cost and net thrust were measured. D) A tear-like foil is used to simplify the fish. The foil is of a length *L* and a head thickness *D* (diameter of the semicircle), where the pitching center is located at the center of the semicircle.

To investigate the general function of small kinematic perturbations to tailbeats during fish-like swimming, we consider perturbations with high frequency and low amplitude compared to a basic sinusoidal wave motion performed by the tail (Fig. 1A and B).

Three modes are defined: basic mode (BM) with a sinusoidal wave representing rhythmic tailbeats (Fig. 1A), perturbation mode (PM) that consists of high-frequency and low-amplitude oscillations (Fig. 1A), and accumulated mode (AM), which represents the combination of sinusoidal wave (BM) and perturbations (PM) (Fig. 1B):


(1) $$\begin{array}{rl} \theta_{\mathrm{bm}}(t)\;= & \Theta_{\mathrm{bm}}\sin\;(2\pi f_{\mathrm{bm}}t)\;\text{BM},\\ \theta_{\mathrm{pm}}(t)\;= & \Theta_{\mathrm{pm}}\sin\;(2\pi f_{\mathrm{pm}}t)\;\text{PM},\\ \theta_{\mathrm{am}}(t)\;= & \theta_{\mathrm{bm}}(t)+\theta_{\mathrm{pm}}(t)\;\text{AM}, \end{array}$$

where subscripts bm, pm, and am refer to the parameters in basic mode (BM), perturbation mode (PM), and accumulated mode (AM), respectively. $\Theta_{\mathrm{bm}}$ and $\Theta_{\mathrm{pm}}$ are the foil’s oscillation angle amplitude in the BM and PM, respectively. $f_{\mathrm{bm}}$ and $f_{\mathrm{pm}}$ refer to the oscillating frequency in the BM and PM, respectively, and *t* is the instantaneous time. To control the frequency and amplitude of the perturbation, we introduce dimensionless frequency $\;\;\tilde{f}=f_{\mathrm{pm}}/f_{\mathrm{bm}}$ and amplitude $\tilde{A}=A_{\mathrm{pm}}/A_{\mathrm{bm}}\approx \Theta_{\mathrm{pm}}/\Theta_{\mathrm{bm}}$ (see Materials and methods for details), respectively. The Strouhal number ratio is $\tilde{St}={St}_{\mathrm{pm}}/{St}_{\mathrm{bm}}$, where ${St}_{\mathrm{bm}}=2f_{\mathrm{bm}}A_{\mathrm{bm}}/U$ and ${St}_{\mathrm{pm}}=2f_{\mathrm{pm}}A_{\mathrm{pm}}/U$ represent the BM-based and PM-based Strouhal numbers, respectively.

Experiments were performed with a robotic fish swimming in a flow tank that was tethered to a high-resolution force sensor. The robotic fish consisted of a high-fidelity goldfish-like body that was capable of replicating various tailbeat kinematics (see Materials and methods; Fig. 1C). We established the basic tailbeat mode with a frequency of $f_{\mathrm{bm}}=1$ Hz and amplitude of $\Theta_{\mathrm{bm}}=0.349$ rad, resulting in a Strouhal number of 0.32, similar to the Strouhal number employed by swimming fish (24–27). The corresponding Reynolds number Re=UL/ν is 1,900, where *L* is the body length and *ν* is the kinematic viscosity of the fluid at a temperature of 20 ^∘^. In the experiments, we measured time-averaged net thrust $\bar{T}$, time-averaged power cost $\bar{P}$, and swimming (Froude) efficiency *η* (= $\bar{T}U/\bar{P}$) of the robotic fish in the BM and AM modes. The power cost, generated thrust, and efficiency ratios with perturbation to those without perturbation are defined as


(2) $$\begin{array}{rl} \tilde{P}\;= & ({\bar{P}}_{\mathrm{am}}-{\bar{P}}_{\mathrm{bm}})/{\bar{P}}_{\mathrm{bm}}\;\text{dimensionless power},\\ \tilde{T}\;= & ({\bar{T}}_{\mathrm{am}}-{\bar{T}}_{\mathrm{bm}})/{\bar{T}}_{\mathrm{bm}}\;\text{dimensionless thrust},\\ \tilde{\eta }\;= & (\eta_{\mathrm{am}}-\eta_{\mathrm{bm}})/\eta_{\mathrm{bm}}\;\text{dimensionless efficiency} \end{array}$$

to quantitatively compare the performances under different modes.

**Fig. 2.**
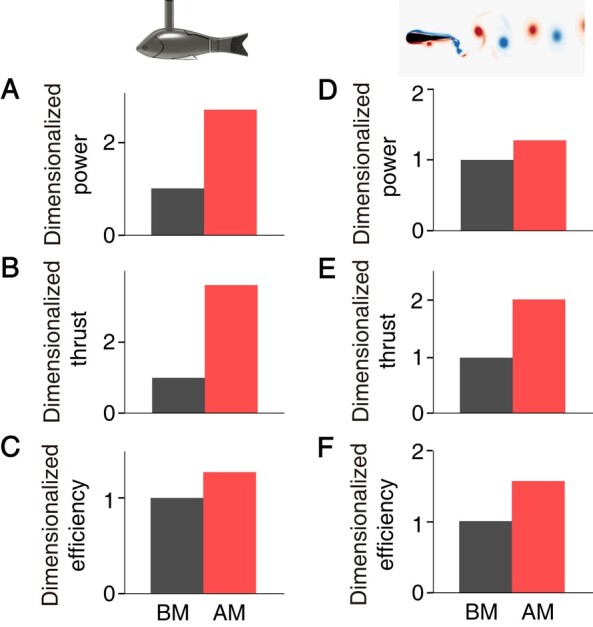
A–C) Dimensionless time-averaged power, thrust, and efficiency of the robotic fish actuated in the BM and AM mode, respectively, where $\left(\;\;\tilde{f},\tilde{A},Re)=(4,0.3,1,900\right)$. D–F) Dimensionless time-averaged power, thrust, and efficiency of the numerical foil actuated in the BM and AM mode, respectively, where $\left(\;\;\tilde{f},\tilde{A},Re)=(10,0.04,1,000\right)$. For both experimental and numerical data, the quantities are normalized by the value produced by the BM mode.

**Fig. 3.**
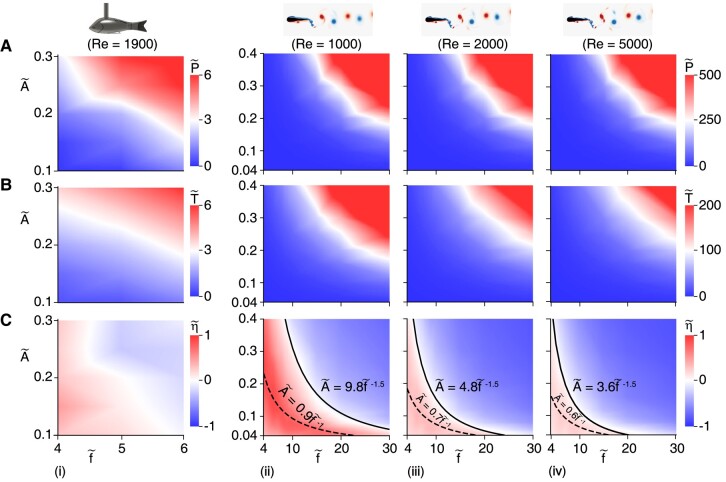
Contours of A) $\tilde{P}$ , B) $\tilde{T}$, and C) $\tilde{\eta }$ in the $\;\;\tilde{f}$– $\tilde{A}$ plane. (i) Experimental results at Re=1,900. (ii–iv) Simulation results at Re=1,000,2,500,5,000, respectively. In panel C(ii–iv), the dashed lines denote the fitting lines for the optimal $\tilde{\eta }$, where the coefficient of determination $R^{2}=0.962$, 0.920, and 0.882, correspond to $\tilde{A}=0.9\;\;{\tilde{f}}^{\;-1.0}$, $\tilde{A}=0.7\;\;{\tilde{f}}^{\;-1.0}$, and $\tilde{A}=0.6\;\;{\tilde{f}}^{-1.0}$, respectively; the solid lines denote the fitting lines for $\tilde{\eta }=0$, where $R^{2}=0.987,0.997$, and 0.983 correspond to $\tilde{A}=9.8\;\;{\tilde{f}}^{\;-1.5}$, $\tilde{A}=4.8\;\;{\tilde{f}}^{\;-1.5}$, and $\tilde{A}=3.6\;\;{\tilde{f}}^{\;-1.5}$, respectively. In simulations, $\eta_{\mathrm{bm}}=9.23,15.04$, and 17.69% correspond to Re=1,000,2,500, and 5,000, respectively.

Experiments were conducted with a perturbation of $\left(\;\tilde{f},\tilde{A})=(4,0.3\right)$. We find $\tilde{P}=265\%$, $\tilde{T}=366\%$, and $\tilde{\eta }=28\%$ (Fig. 2A–C; Movie S1), suggesting that introducing relatively high-frequency and low-amplitude perturbations to tailbeats can improve both net thrust and swimming efficiency simultaneously. To systematically explore how perturbation influences the fish propulsion, we further studied the function of perturbations with $\;\;\tilde{f}$ ranging from 4 to 6 and $\tilde{A}$ ranging from 0.1 to 0.3. As shown in Fig. 3A and B(i), both $\tilde{P}$ and $\tilde{T}$ are positively correlated with $\;\;\tilde{f}$ and/or $\tilde{A}$. This reveals that the stronger the perturbations, the higher the power costs, and the higher the generated net thrusts. Moreover, the normalized net thrust $\tilde{T}$ are all above 0, indicating that perturbations always improve net thrust. In contrast, Fig. 3C(i) indicates that the normalized efficiency $\tilde{\eta }$ has a complex relationship as a function of $\;\;\tilde{f}$ and $\tilde{A}$. In general, with fixed $\;\;\tilde{f}$ ($\tilde{A}$), an improvement of $\tilde{A}$ ($\;\tilde{f}$) will first improve and then inhibit $\tilde{\eta }$.

### Simulations on pitching foil

To explore the parameter space beyond the range of experimental capabilities, especially with respect to large variations in perturbations ($\;\tilde{f}$ and $\tilde{A}$) that would impose high torques on the actuators, we established a computational fluid dynamics simulation environment using FLUENT (ANSYS version 14.0). The simulations also enable a more comprehensive analysis of the flow profiles that result from superimposed perturbations and thus shed light on the hydrodynamic features underlying the observed trends. For simulations, we employed a simplified tear-like foil with the dimensionless thickness of D/L=0.196 (*D* denotes foil thickness, Fig. 1D).

To validate the simulation model with respect to physical experiments, we conducted an investigation of the swimming performance of a pitching foil with a perturbation of $\left(\;\tilde{f},\tilde{A})=(10,0.04\right)$ and a basic wave with frequency $f_{\mathrm{bm}}=2$ Hz and amplitude $\Theta_{\mathrm{bm}}=0.175$ rad at a Reynolds number of $10^{3}$. In our simulations, we also observed that adding perturbations enhances both thrust and efficiency ($\tilde{P}$ = 27%, $\tilde{T}$ = 101%, and $\tilde{\eta }$ = 58%), consistent with our experimental findings (Fig. 2D–F; Movie S2).

To systematically explore the impact of perturbations over a wider range of parameters, we conducted simulations by varying $\;\;\tilde{f}$ ranging from 4 to 30, $\tilde{A}$ ranging from 0.04 to 0.4, for Reynolds numbers of 1,000, 2,500, and 5,000. Figure 3A–C(ii–iv) summarizes the results after over 80,000 core hours of simulations. We systematically explored the normalized power cost, net thrust, and swimming efficiency as a function of normalized frequency $\;\;\tilde{f}$ and amplitude $\tilde{A}$. With increasing in $\;\;\tilde{f}$ and/or $\tilde{A}$, $\tilde{P}$, and $\tilde{T}$ gradually increase, while $\tilde{\eta }$ first increases and then decreases. As expected, the numerical results conform to those obtained from the experiments, with deviations occurring due to features such as the difference in the profile of baseline tailbeat kinematics, differences in morphology, and differences in dimensions. The similarity in the trends of the improvement in efficiency noticed in both simulations and experiments suggests that the benefits of perturbations are robust and, therefore, could be generic. The optimal case of simulations is obtained at $\left(\;\tilde{f},\tilde{A},Re)=(4,0.25,10^{3}\right)$, resulting in $\left(\tilde{P},\tilde{T},\tilde{\eta })=(196\%,467\%,92\%\right)$. Additionally, optimal lines for $\tilde{\eta }$ are formed as $\tilde{A}∼\;\;{\tilde{f}}^{\;-1}$, indicating that significant improvement in $\tilde{\eta }$ could be achieved through high-frequency, low-amplitude perturbations and vice versa. Moreover, our numerical results also show that while all perturbations enhance thrust, not all perturbations improve efficiency. Using the power fitting method, we found that the lines formed as $\tilde{A}∼\;\;{\tilde{f}}^{\;-1.5}$ (coefficient of determination $R^{2}>0.98$) distinguish between improvement and decline in efficiency.

### Wake structures and moment analysis

**Fig. 4.**
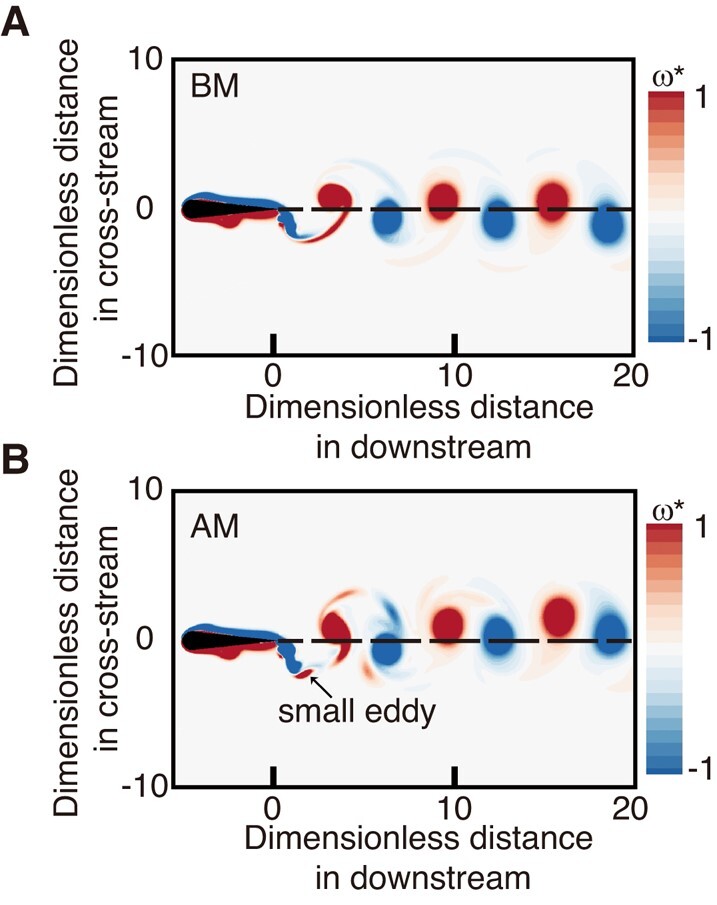
Flow patterns of the foil in A) BM and B) AM, where the red and blue colors denote positive and negative vorticities, respectively. $\omega^{*}=\omega D/U$, where *ω* is the spanwise vorticity $\left(\;\tilde{f},\tilde{A},Re)=(10,0.04,1,000\right)$.

To understand the interaction between pitching foil and flow induced by superimposed high-frequency and low-amplitude perturbations, we further studied the wake structures provided by both BM and AM to visualize the complex dynamics of fluids. At $tf_{\mathrm{bm}}=18$, we illustrated the contour of vorticity $\omega^{*}=\omega D/U$ using the numerical simulations results at $\left(\;\tilde{f},\tilde{A},Re)=(10,0.04,10^{3}\right)$, where *ω* is the spanwise vorticity. As shown in Fig. 4A, the basic mode (BM) generates 2S wakes (39), a typical reverse Bénard–von Kármán (rBvK) vortex street, leading to a jet-flow (Fig. S2A). In contrast, accumulated mode (AM) generated additional smaller eddies due to superimposing the perturbations (Fig. 4B), resulting in a deflected flow which slants upward first and then downward (Fig. S2B).

There are two stages during the vortex shedding: vortex development (VD) and vortex transportation (VT). VD, viewed as a precursor to VT, physically describes the process of vorticities feeding from the shear layer and is terminated when the generated vortex can no longer entrain additional vorticity from the vortex generator (referred to as the vortex pinchoff) (40, 41). The vortex pinchoff occurs when the vortex possesses maximum energy from a vortex generator (42), or the vortex generator suddenly stops the energy injection into the vortex, such as the flapping oscillation considered in present work (43). The superimposed perturbations cause the foil to alter its flapping direction rapidly, and multiple vortex pinchoffs occur. As a result, the small-scale eddies provided by perturbations in the wake will be separated between the large-scale vortices generated by the basic oscillation, leading to variations in the positioning of dominant vortices (Fig. S3).

**Fig. 5.**
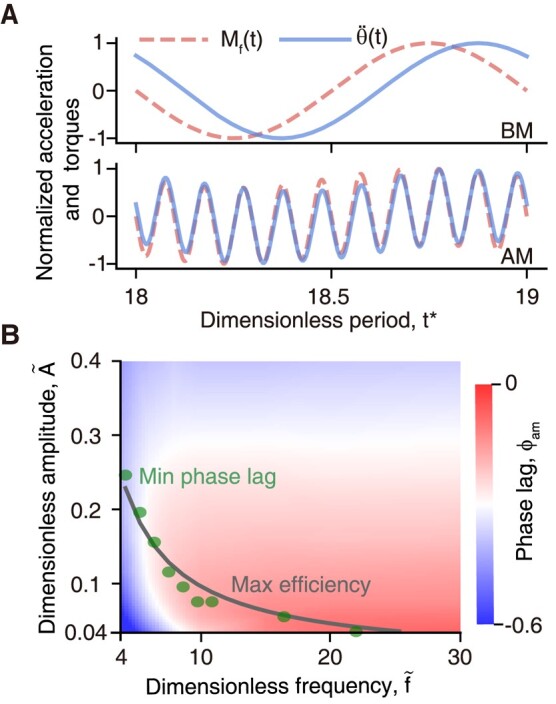
A) Normalized foil pitching acceleration $\ddot{\theta }(t)$ and flow-induced torque $M_{f}(t)$ in BM and AM, respectively, where $(\;\tilde{f},\tilde{A},Re)\;=$  (10,0.04,1,000). B) The $\phi_{\mathrm{am}}$ contour in $\;\;\tilde{f}-\tilde{A}$ plane at Re=1,000, where the solid line refers to the optimal efficiency improvement line in Fig. 3C(ii) and the circle symbol denotes the location of the minimal $\left|\phi_{\mathrm{am}}\right|$ at the specific $\;\;\tilde{f}$.

Since the time-averaged power cost can be calculated as $\bar{P}=f_{\mathrm{bm}}\int_{0}^{1/f_{\mathrm{bm}}}M_{f}(t)\dot{\theta }(t)\text{d}t$ (see Materials and methods for details), where $M_{f}(t)$ is the torque induced by fluid dynamics, it is worth understanding how $M_{f}(t)$ is affected by superimposed perturbations to demystify the mechanisms underlying efficiency improvements. Figure 5A reveals the $M_{f}(t)$ provided by BM remains the same frequency with $\ddot{\theta }(t)$, while the perturbation dominates $M_{f}(t)$ profile in AM. Particularly, a phase lag *ϕ* exists between $\ddot{\theta }(t)$ and $M_{f}(t)$. This *ϕ* described the phase lag between foil’s oscillation and motion-induced fluid dynamics response is actually sourced from the “memory effect” in flow (44). Using the Hilbert transform, we calculated $\phi_{\mathrm{bm}}$ and $\phi_{\mathrm{am}}$ referred to BM-based and AM-based phase lag, respectively, and found $\left|\phi_{\mathrm{am}}|<|\phi_{\mathrm{bm}}\right|$. This discrepancy is attributed to the high-frequency components introduced by superimposed perturbations, which induce rapid transitions in pitching velocity, accelerate the diffusion–convection process in the vorticity layer created on the foil’s surface after a change in velocity, and thus facilitate an efficient response of the fluid dynamics to the pitching oscillation.

Mathematically, the minimal $\bar{P}$ will be achieved when $M_{f}(t)$ and $\dot{\theta }(t)$ are out-of-phase, which corresponds to $M_{f}(t)$ and $\ddot{\theta }(t)$ being in-phase, i.e. ϕ=0. On the other hand, a smaller phase lag between fluid and flow-driven motion is thought to be beneficial for enhancing the energy harvesting efficiency in a flow-induced vibration system (45). Figure 5B implies that $\phi_{\mathrm{am}}$ is a function of $\;\;\tilde{f}$ and $\tilde{A}$ and the minimal $\left|\phi_{\mathrm{am}}\right|$ values agree well with the data-driven optimal $\tilde{\eta }$ line in Fig. 3C(ii). This result reveals that phase lag is a critical parameter in affecting efficiency production, a smaller *ϕ* would cause less energy consumption and potentially improve swimming efficiency.

### Analytical models

To explore the mechanisms underlying the improvements in propulsive thrust and swimming efficiency, we further presented analytical models to examine the correlation between dimensionless thrust and efficiency, and the dimensionless frequency and amplitude.

The time-averaged thrust acting on the foil $\bar{T}$ is the sum of the time-averaged pressure-induced thrust ${\bar{T}}^{P}(>0)$ and time-averaged viscous drag ${\bar{T}}^{V}(<0)$, as


(3) $$\bar{T}={\bar{T}}^{P}+{\bar{T}}^{V}.$$

Inspired by previous works on pitching foils (31, 46, 47), the time-averaged pressure-induced thrusts read as


(4) $$\begin{array}{cc} & {\bar{T}}_{\mathrm{bm}}^{P}∼\rho U^{2}S_{f}{St}_{\mathrm{bm}}^{2}\;\text{in BM},\\ & {\bar{T}}_{\mathrm{am}}^{P}∼\rho U^{2}S_{f}({St}_{\mathrm{bm}}^{2}+{St}_{\mathrm{pm}}^{2})\;\text{in AM}, \end{array}$$

where $S_{f}$ denotes the surface area of the foil, while


(5) $$\begin{array}{cc} & {\bar{T}}_{\mathrm{bm}}^{V}∼-\rho U^{2}S_{f}Re^{-\frac{1}{2}}\sqrt{{St}_{\mathrm{bm}}}\;\text{in BM},\\ & {\bar{T}}_{\mathrm{am}}^{V}∼-\rho U^{2}S_{f}Re^{-\frac{1}{2}}\sqrt{{St}_{\mathrm{bm}}+{St}_{\mathrm{pm}}}\;\text{in AM} \end{array}$$

can be driven from the Bone–Lighthill boundary-layer thinning hypothesis (48, 49). Equation 5 reveals the magnitude of viscous drag produced by the AM is slightly larger than that produced by the BM (${\bar{T}}_{\mathrm{am}}^{V}/{\bar{T}}_{\mathrm{bm}}^{V}∼\sqrt{1+\tilde{St}}$). At $\left(\;\;\tilde{f},\tilde{A},Re)=(10,0.04,10^{3}\right)$, we found ${\bar{T}}_{\mathrm{am}}^{V}/{\bar{T}}_{\mathrm{bm}}^{V}>1$ (Fig. S4), agreeing with the theoretical prediction. The increase of viscous drag undergoing the AM is owed to the compression of the shear layer and the acceleration of the tangential component of the potential outer flow near the foil. With increasing in *Re*, ${\bar{T}}^{V}$ approaches zero (Eq. 5), suggesting the pressure-induced thrust dominates the thrust generation (50, 51). We thus have


(6) $$\tilde{T}∼{\tilde{St}}^{2}$$

when the viscous effect is neglected (Re→∞). As the perturbations introduce the dimensionless amplitude and frequency, the Strouhal number ratio $\tilde{St}$ also increases ($\tilde{St}>0$), and thus time-averaged thrust is improved ($\tilde{T}>0$, Fig. 3B). Equation 6 further reveals the thrust enhancement in AM is mainly sourced from the increase of pressure-induced thrust (Fig. S4).

We next derive the dimensionless efficiency as a function of the dimensionless frequency and amplitude. To do so, we also need to estimate dimensionless power cost, as


(7) $$\begin{array}{rl} \bar{P} & =f_{\mathrm{bm}}\int_{0}^{1/f_{\mathrm{bm}}}\left[M_{i}(t)-M_{f}(t)\right]\times \dot{\theta }(t)\;\text{d}t\\ & =f_{\mathrm{bm}}\int_{0}^{1/f_{\mathrm{bm}}}M_{f}(t)\dot{\theta }(t)\;\text{d}t, \end{array}$$

where $M_{i}(t)=J\ddot{\theta }(t)$ is the inertia torque with the mass moment of inertia of the foil *J*, the $M_{f}(t)$ is calculated as $M_{f}(t)=-c_{1}\ddot{\epsilon }(t)-c_{2}U\dot{\epsilon }(t)$ with two positive coefficients $c_{1}∼L^{4}$ and $c_{2}∼L^{3}$ (*L* represents body length), $\dot{\epsilon }(t)=\dot{\theta }(t+\phi )$ and $\ddot{\epsilon }(t)=\ddot{\theta }(t+\phi )$ refer to the angular velocity and acceleration of the fluid around the foil, respectively (46). Then, we have the $\tilde{P}$ scaling as (see Materials and methods for details)


(8) $$\tilde{P}∼\frac{\left({\tilde{A}}^{-1}{\tilde{St}}^{3}+1)\sin\;(\phi_{\mathrm{am}}\right)}{\sin\;(\phi_{\mathrm{bm}})}-1$$

when $A_{\mathrm{pm}}\approx 0$ is caused by the low-amplitude pitching.

As a result, we obtain the efficiency $\tilde{\eta }$ scaling as (see Materials and methods for details)


(9) $$\tilde{\eta }∼\frac{\left({\tilde{St}}^{2}+1)\sin\;(\phi_{\mathrm{bm}}\right)}{\left({\tilde{A}}^{-1}{\tilde{St}}^{3}+1)\sin\;(\phi_{\mathrm{am}}\right)}-1$$

when Re→∞ and $A_{\mathrm{pm}}\to 0$. Equation 9 reveals a smaller $\phi_{\mathrm{am}}$ would lead to a larger $\tilde{\eta }$ with a special $\tilde{St}$ and $\tilde{A}$.

**Fig. 6.**
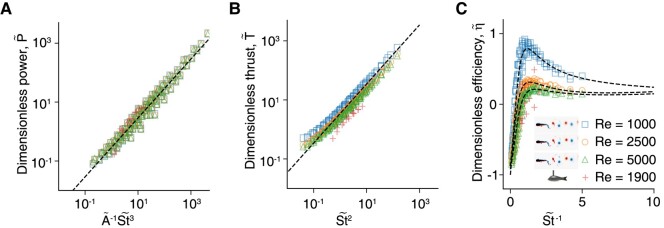
Scaling for the dimensionless A) power $\tilde{P}$, B) thrust $\tilde{T}$, and C) efficiency $\tilde{\eta }$. Dashed lines refer to the scaling lines derived from Eqs. 8, 6, and 9, respectively.

The analytical models are verified by comparing to both experimental and simulation results in Fig. 6. The scaling equations degenerate as $\tilde{T}=0$, $\tilde{P}=0$ and $\tilde{\eta }=0$, respectively, when no perturbations have been added ($\tilde{St}=0$, and $\phi_{\mathrm{bm}}$ = $\phi_{\mathrm{am}}$).

## Discussion and conclusion

Swimming is energetically expensive and animals and robots need efficient kinematic strategies to increase the thrust produced and maximize their locomotion efficiency. This study investigates the impact of introducing kinematic perturbations to the rhythmic tailbeat of fish-like swimming oscillators. By systematically varying the profile of the perturbation in relation to the kinematics, we find that superimposing perturbations consistently increase thrust but not swimming efficiency. Given that only particular perturbations enhance both thrust and efficiency, our study suggests that previous conflicting results may arise from variations in parameter spaces (37, 38).

The mechanism we found for improving efficiency complements previous studies. Prior research has suggested that the optimal swimming efficiency is determined by the fluid drag on fins and wings, with a lower drag resulting in a higher efficiency (52). However, in our case, we found that superimposing perturbations leads to higher viscous drag but still improves efficiency (Eq. 5; Fig. S4). Another previously proposed way to enhance swimming efficiency is to increase the maximal angle of attack (AoA) below the dynamic stall limit, which prevents the shedding of leading-edge vortices (52, 53). In our study, perturbations, particularly those with small amplitudes, do not noticeably enhance the maximal AoA (Eq. 1); therefore, the efficiency gain from perturbations is not attributable to increased maximal AoA. Additionally, the efficiency improvement may be due to resonance between the natural frequency of the oscillating system and the frequency of vortex shedding (54). We cannot reject the hypothesis corresponding to the resonance effects since reducing the phase lag between body motion and fluid dynamics responses also affects the frequency of vortex shedding.

According to Granger and Paidoussis (44), the phase lag is likely caused by the flow retardation effect, which results in a reorganization of the flow around the body, leading to a time delay between body motion and fluid dynamics response, known as the phase lag. This is different from the time delay caused by the Wagner effect (55) (also known as the “start vortex effect”) as the phase lag maintains after the initialization. As the Reynolds number increases, the boundary layer becomes thinner, which in turn diminishes the rate of acceleration for the diffusion–convection of the vorticity. This leads to a smaller reduction of phase lag and thus lower efficiency improvement. Therefore, the higher the Reynolds number, the smaller the improvement in efficiency. Our findings in Fig. 6C are consistent with this, as we observe that the efficiency improvement from certain superimposed perturbations becomes insignificant at higher Reynolds numbers. On the other hand, as *Re* increases, it would result in a reduction in efficiency improvement since the swimming efficiency provided by the BM increases with increasing in *Re* (50). This further indicates that superimposed perturbations to improve locomotion may be more beneficial for larval fish than adult fish, since the viscosity has a greater impact on larvae (56). These findings could also explain why larval fish exhibit more random body waves while adults exhibit clearer stereotypical sinusoidal tailbeat kinematics (57).

Further examination of the impact of more general perturbations on thrust and efficiency will be beneficial. Specifically, investigating the effects of nonkinematic higher frequency and lower amplitude random perturbations, which more accurately replicate the perturbations that animals experience in the wild, such as those caused by turbulence during flight or swimming, will be useful. The findings and analytical models presented here lay the foundation for extending to practical applications that can effectively introduce kinematic perturbation to deliver thrust and efficiency improvements to general underwater robots or gliders.

## Materials and methods

### Experimental setup and procedure

Figure 1C shows a schematic of the experimental setup. The experiments were conducted at the Max Planck Institute of Animal Behavior using a flow tank with dimensions of 250×875×250 millimeters (mm) in width, length, and depth. The flow speed was calibrated to be linearly proportional to the control voltage. Our robotic fish models were designed according to the morphology of the goldfish with a body length of 15 cm. We first scanned real fish to obtain the 3D fish body as a point cloud map. Reverse modeling of the mesh from the cloud map was utilized for our mechanical design using SolidWorks, and the fish body was printed with a 3D printer (Ultimaker S3). Limited by the size of the real fish, we included a single joint in this robot design. The oscillation of this joint is controlled by a waterproof servomotor (Hitec HS-5086WP) driven by a central pattern generator control (58).

Thrust T(t) was measured using a force balance with a full-scale range of 5 kg (Z6FD1, Deutschland HBM). Power P(t) was calculated as the input energy of the motion-generated motor. Data were sampled at a rate of 5,000 points per second using a current acquisition system (NI-9227, National Instruments). Each experimental trial consisted of a 5-s initialization period, during which the robotic fish swam to establish the vortex stream, followed by a 5-s data collection period. To ensure reliability, each set of parameters was repeated five times. For the base mode, we find $P_{\mathrm{bm}}=0.775\;W$, $T_{\mathrm{bm}}=0.183\;N$, $\eta_{\mathrm{bm}}=T_{\mathrm{bm}}/P_{\mathrm{bm}}=0.236\;N/W$.

### Simulations

In our study, we adopted the mesh system and numerical method previously used by Muhammad et al. (35). The computational domain used in the simulations is shown in Fig. S6. The foil’s geometry was defined using the same parameters as in Fig. 1D. The computational domain is a 2D rectangular region with dimensions of 200D×100D, where *D* is the foil thickness. The inlet velocity boundary condition is located at a distance of 50D from the pitching center of the foil, with a velocity vector (U,0). The outflow boundary is situated 150D downstream, with no-stress outflow boundary conditions. The upper and lower boundaries are slip walls located 50D apart from the foil. The computational domain is divided into three grid zones: Zone 1, Zone 2, and Zone 3. Zone 1, inside the blue circle region, has a high grid resolution around the foil. Zone 1 is given an O-xy mesh system where the grids move with the foil as a rigid body. In Zone 1, the first cell is placed at a distance of 0.004D from the foil surface. Zone 2, the black region, has a high resolution to capture large velocity gradients. The blue circle is a circular nonconformal sliding grid interface. It is the interface between Zone 1 and Zone 2. Zone 3, located away from the wake region, has a medium resolution. Zones 2 and 3 employ structured meshes. The prescribed motion of Eq. 1 is controlled using a user defined function with the DEFINE _CG _MOTION function.

The grid and time-step independence tests were performed to validate the numerical simulations at the most strenuous parameters $\left(\;\tilde{f},\tilde{A})=(30,0.40\right)$ with Reynolds number of $Re=5\times 10^{3}$. Figure S7 shows the results. The grid independence test is done for three sets of grids with $1.14\times 10^{5}$ (G1), $2.13\times 10^{5}$ (G2), and $4.18\times 10^{5}$ (G3) corresponding to 233, 350, and 525 points on the foil surface, respectively. The time-step of $\Delta t=1/(2,000f_{\mathrm{pm}})$ in the grid independence test is decided by Muhammad et al. (35) and the empirical formula ($\Delta t=\min\{1/(2,000f_{\mathrm{pm}}),L/\|V\|\}$, where ‖V‖ is the maximal instantaneous convective flux velocity in the computational domain) proposed by Kinsey and Dumas (59). The dimensionless instantaneous thrust (by ${\bar{T}}_{\mathrm{bm}}$) over one pitching period ($1/f_{\mathrm{bm}}$) for the three grid systems show no obvious difference is observed between G2 and G3 (Fig. S7A and Table S1). The difference in the dimensionless time-averaged thrust (by ${\bar{T}}_{\mathrm{bm}}$) between G2 and G3 is reasonably small, as 0.65%. Mesh G2 is thus adopted. With G2, three time-steps $\Delta t_{1}=1/(1,000f_{\mathrm{pm}})$, $\Delta t_{2}=1/(2,000f_{\mathrm{pm}})$, and $\Delta t_{3}=1/(4,000f_{\mathrm{pm}})$ are tested. The result of dimensionalized instantaneous thrust reveals the difference in the dimensionless time-averaged thrust between $\Delta t_{2}$ and $\Delta t_{3}$ is only 0.53% (Fig. S7B and Table S2). Considering the accuracy and computational resources, $\Delta t_{2}$ is chosen for the extensive work.

In our simulation, the simulation is performed using a grid number of $2.13\times 10^{5}$, corresponding to 350 points on the foil surface, and a time-step $\Delta t=1/(2,000f_{\mathrm{pm}})$. To ensure statistically steady thrust and wake structures, all simulations are run for 20 pitching periods ($1/f_{\mathrm{bm}}$), and the averages are calculated over the final 5 simulation periods after achieving statistical convergence. For the base mode, we find ($P_{\mathrm{bm}}/0.5\rho U^{3}L$, $T_{\mathrm{bm}}/0.5\rho U^{2}L$, $\eta_{\mathrm{bm}}$ = 0.068, 0.006, 0.092) at Re=1,000, ($P_{\mathrm{bm}}/0.5\rho U^{3}L$, $T_{\mathrm{bm}}/0.5\rho U^{2}L$, $\eta_{\mathrm{bm}}$ = 0.071, 0.011, 0.15) at Re=2500, and ($P_{\mathrm{bm}}/0.5\rho U^{3}L,$  $T_{\mathrm{bm}}/0.5\rho U^{2}L,$  $\eta_{\mathrm{bm}}$ = 0.072, 0.013, 0.177) at Re=5,000, where $\eta_{\mathrm{bm}}=T_{\mathrm{bm}}U/P_{\mathrm{bm}}$, respectively.

### Models

In Eq. 3, ${\bar{T}}^{P}$ reads as


(10) $${\bar{T}}^{P}∼f_{\mathrm{bm}}\int_{0}^{1/f_{\mathrm{bm}}}\rho S_{f}\times {\dot{\epsilon }}^{2}(t)\times \text{cos}\left[\theta (t)\right]\;\text{d}t.$$

Here, $S_{f}$ denotes the surface area of the foil, $\dot{\epsilon }(t)$ is the angular velocity of the fluid around the foil. As introduced before, a phase lag *ϕ* exits between θ(t) and ϵ(t), we thus have


(11) $$\begin{array}{rl} {\dot{\epsilon }}_{\mathrm{bm}}(t) & =2\pi f_{\mathrm{bm}}\Theta_{\mathrm{bm}}\text{cos}\left(2\pi f_{\mathrm{bm}}t+\phi_{\mathrm{bm}}\right),\\ {\dot{\epsilon }}_{\mathrm{am}}(t) & =2\pi [f_{\mathrm{bm}}\Theta_{\mathrm{bm}}\text{cos}\left(2\pi f_{\mathrm{bm}}t+\phi_{\mathrm{am}}\right)\\ & \;+f_{\mathrm{pm}}\Theta_{\mathrm{pm}}\text{cos}\left(2\pi f_{\mathrm{pm}}t+\phi_{\mathrm{am}}\right)] \end{array}$$

corresponding to BM and AM, respectively. Considering a small $\Theta_{\mathrm{bm}}$ and even smaller $\Theta_{\mathrm{pm}}$, we get $\text{cos}[\theta_{\mathrm{bm}}(t)]\approx \text{cos}[\theta_{\mathrm{am}}(t)]\approx 1$ (Fig. S8), and $\Theta_{\mathrm{pm}}/\Theta_{\mathrm{am}}\approx A_{\mathrm{pm}}/A_{\mathrm{bm}}=\tilde{A}$. Therefore, the time-averaged pressure thrusts read as Eq. 4.

The time-averaged viscous drag can be estimated using results of the works on the Bone–Lighthill boundary-layer thinning hypothesis (48, 49), as Eq. 5.

We thus have


(12) $$\tilde{T}∼\frac{{\tilde{St}}^{2}-\frac{1}{c_{0}}{St}_{\mathrm{bm}}^{-\frac{3}{2}}Re^{-}\frac{1}{2}(\sqrt{\tilde{St}+1}-1)}{1-\frac{1}{c_{0}}{St}_{\mathrm{bm}}^{-\frac{3}{2}}Re^{-\frac{1}{2}}}$$

with a positive constant $c_{0}$. Particularly, when the viscous effect is neglected (Re→∞), Eq. 12 degenerates as Eq. 6.

We next derive the dimensionless efficiency as a function of the dimensionless frequency and amplitude. The time-averaged power cost is considered as the product of the net torque $M(t)=M_{i}(t)-M_{f}(t)$ and $\ddot{\theta }(t)$ on the pitching foil, i.e. as introduced before, we get Eq. 7.

For BM and AM, the net torque M(t) can be calculated as


(13) $$\begin{array}{rl} M_{\mathrm{bm}}(t) & =-(4\pi^{2})Jf_{\mathrm{bm}}^{2}\Theta_{\mathrm{bm}}\sin\;(2\pi f_{\mathrm{bm}}t)\\ & \;-(4\pi^{2})c_{1}f_{\mathrm{bm}}^{2}\Theta_{\mathrm{bm}}\sin\;(2\pi f_{\mathrm{bm}}t+\phi_{\mathrm{bm}})\\ & \;+(2\pi )c_{2}Uf_{\mathrm{bm}}\Theta_{\mathrm{bm}}\text{cos}(2\pi f_{\mathrm{bm}}t+\phi_{\mathrm{bm}}), \end{array}$$

and


(14) $$\begin{array}{rl} M_{\mathrm{am}}(t) & =-(4\pi^{2})J[f_{\mathrm{bm}}^{2}\Theta_{\mathrm{bm}}\sin\;(2\pi f_{\mathrm{bm}}t)\\ & \;+f_{\mathrm{pm}}^{2}\Theta_{\mathrm{pm}}\sin\;(2\pi f_{\mathrm{pm}}t)]\\ & \;-(4\pi^{2})c_{1}[f_{\mathrm{bm}}^{2}\Theta_{\mathrm{bm}}\sin\;(2\pi f_{\mathrm{bm}}t+\phi_{\mathrm{am}})\\ & \;+f_{\mathrm{pm}}^{2}\Theta_{\mathrm{pm}}\sin\;(2\pi f_{\mathrm{pm}}t+\phi_{\mathrm{am}})]\\ & \;+(2\pi )c_{2}U[f_{\mathrm{bm}}\Theta_{\mathrm{bm}}\text{cos}(2\pi f_{\mathrm{bm}}t+\phi_{\mathrm{am}})\\ & \;+f_{\mathrm{pm}}\Theta_{\mathrm{pm}}\text{cos}(2\pi f_{\mathrm{pm}}t+\phi_{\mathrm{am}})], \end{array}$$

respectively. The $\dot{\theta }(t)$ can be directly obtained from the kinematics (Eq. 1), as


(15) $$\begin{array}{cc} & {\dot{\theta }}_{\mathrm{bm}}(t)=2\pi f_{\mathrm{bm}}\Theta_{\mathrm{bm}}\text{cos}(2\pi f_{\mathrm{bm}}t)\;\text{in BM},\\ & {\dot{\theta }}_{\mathrm{am}}(t)=2\pi [f_{\mathrm{bm}}\Theta_{\mathrm{bm}}\text{cos}(2\pi f_{\mathrm{bm}}t)\\ & \;\;+f_{\mathrm{pm}}\Theta_{\mathrm{pm}}\text{cos}(2\pi f_{\mathrm{pm}}t)]\;\text{in AM}. \end{array}$$

Since $\int_{0}^{1/f_{\mathrm{bm}}}M_{i}(t)\dot{\theta }(t)\text{d}t=\int_{0}^{1/f_{\mathrm{bm}}}J\ddot{\theta }(t)\dot{\theta }(t)\text{d}t=0$. The power cost is actually the integral of the product of $M_{f}(t)$ and $\dot{\theta }(t)$. Through substituting Eqs. 13–15 into Eq. 7, the time-averaged power cost reads as


(16) $$\begin{array}{rl} {\bar{P}}_{\mathrm{bm}}(t)∼ & -c_{3}Lf_{\mathrm{bm}}^{3}\Theta_{\mathrm{bm}}^{2}\sin\;(\phi_{\mathrm{bm}})\\ & +Uf_{\mathrm{bm}}^{2}\Theta_{\mathrm{bm}}^{2}\text{cos}(\phi_{\mathrm{bm}})\;\text{in BM},\\ {\bar{P}}_{\mathrm{am}}(t)∼ & -c_{3}L(f_{\mathrm{bm}}^{3}\Theta_{\mathrm{bm}}^{2}+f_{\mathrm{pm}}^{3}\Theta_{\mathrm{pm}}^{2})\sin\;(\phi_{\mathrm{am}})\\ & +U(f_{\mathrm{bm}}^{2}\Theta_{\mathrm{bm}}^{2}+f_{\mathrm{pm}}^{2}\Theta_{\mathrm{pm}}^{2})\text{cos}(\phi_{\mathrm{am}})\;\text{in AM} \end{array}$$

with the positive coefficient $c_{3}$. Then, we have the $\tilde{P}$ scaling as


(17) $$\tilde{P}∼\frac{-c_{3}L{St}_{\mathrm{bm}}(\tilde{A}+{\tilde{St}}^{3})\sin\;(\phi_{\mathrm{am}})+A_{\mathrm{pm}}(1+{\tilde{St}}^{2})\text{cos}(\phi_{\mathrm{am}})}{-c_{3}L\tilde{A}{St}_{\mathrm{bm}}\sin\;(\phi_{\mathrm{bm}})+A_{\mathrm{pm}}\text{cos}(\phi_{\mathrm{bm}})}-1.$$

Note $A_{\mathrm{pm}}\approx 0$ caused by the low-amplitude pitching, the Eq. 17 can therefore be simplified as Eq. 8.

As a result, we obtain the efficiency $\tilde{\eta }$ scaling as


(18) $$\begin{array}{rl} \tilde{\eta } & =\frac{\tilde{T}+1}{\tilde{P}+1}-1∼\frac{\left({\tilde{St}}^{2}+1)-\frac{1}{c_{0}}{St}_{\mathrm{bm}}^{-\frac{3}{2}}Re^{-\frac{1}{2}}(\sqrt{\tilde{St}+1}\right)}{1-\frac{1}{c_{0}}{St}_{\mathrm{bm}}^{-\frac{3}{2}}Re^{-\frac{1}{2}}}\\ & \;\times \frac{-c_{3}L\tilde{A}\sin\;(\phi_{\mathrm{bm}})+A_{\mathrm{pm}}{St}_{\mathrm{bm}}^{-1}\text{cos}(\phi_{\mathrm{bm}})}{-c_{3}L({\tilde{St}}^{3}+\tilde{A})\sin\;(\phi_{\mathrm{am}})+A_{\mathrm{pm}}{St}_{\mathrm{bm}}^{-1}({\tilde{St}}^{2}+1)\text{cos}(\phi_{\mathrm{am}})}-1. \end{array}$$

In particular, when Re→∞ and $A_{\mathrm{pm}}\to 0$, we get Eq. 9.

## Supplementary Material

Supplementary material is available at *PNAS Nexus* online.

## Supplementary Material

pgae073_Supplementary_Data

## Data Availability

The data that support the findings of this study are available in figshare with the identifier https://doi.org/10.6084/m9.figshare.25272523. Source data are provided with this paper.

## Previous Presentation

These results have not been presented previously.
