# Supplementary material for: Tailbeat perturbations improve swimming efficiency by reducing the phase lag between body motion and the resulting fluid response
Source: PNAS Nexus. 2024 Feb 17;3(3):pgae073. doi: 10.1093/pnasnexus/pgae073 (PMC10939483; doi:10.1093/pnasnexus/pgae073)
Supplement: pgae073_Supplementary_Data [file pgae073_supplementary_data.zip › PNASNEXUS-PNASNEXUS-2023-01012R-s01.pdf]

Supporting Information for

Tailbeat perturbations improve swimming efficiency by reducing the phase lag between body motion and the resulting fluid response

Li-Ming Chao, Laibing Jia, Siyuan Wang, Alexander Liberzon, Sridhar Ravi, Iain D. Couzin and Liang Li

Liang Li and Laibing Jia.
E-mail: lli@ab.mpg.de; l.jia@strath.ac.uk

This PDF file includes:

Figs. S1 to S8
Tables S1 to S2
Legends for Movies S1 to S2

Other supporting materials for this manuscript include the following:

Movies S1 to S2

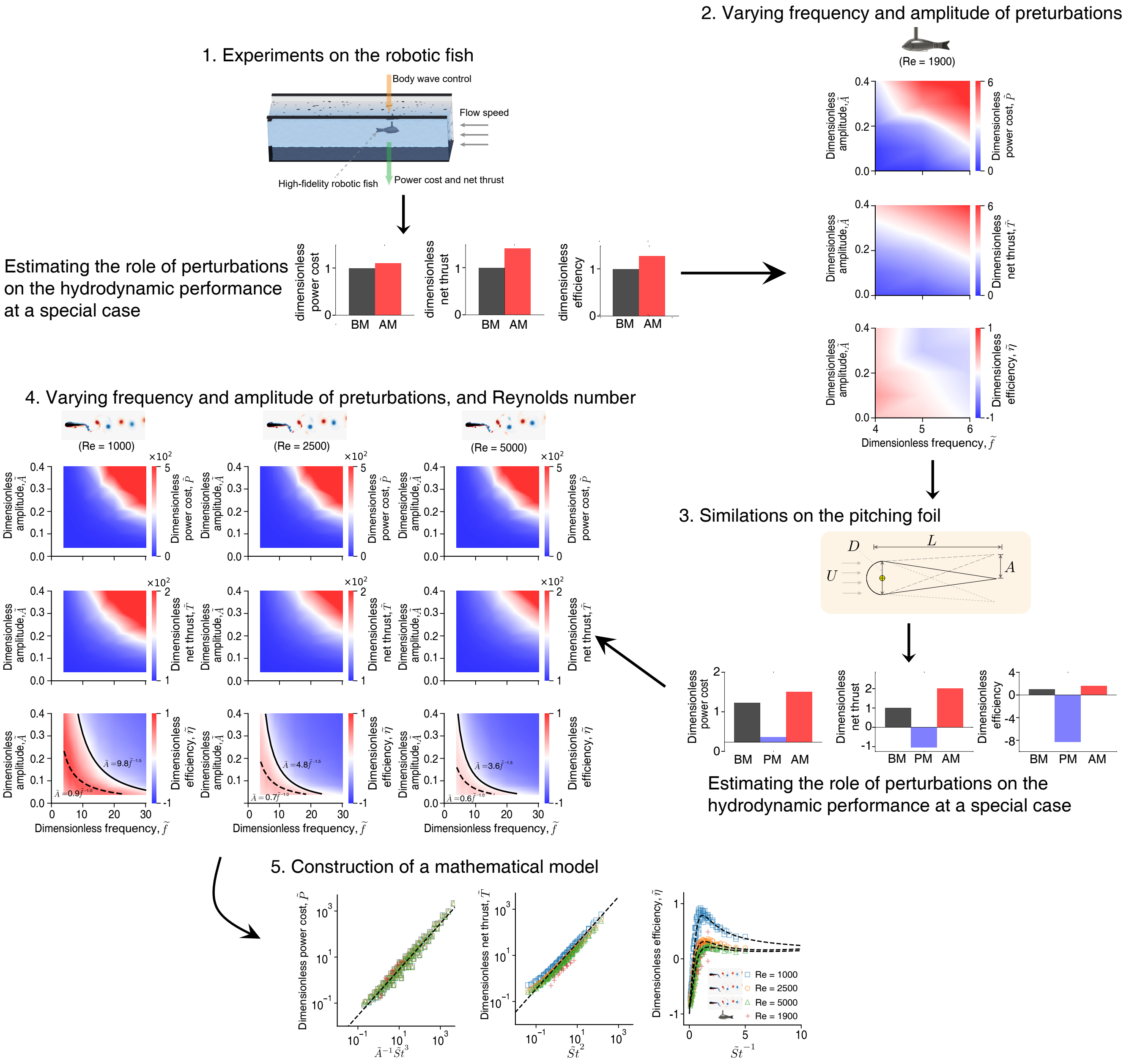

Fig. S1. Schematic of the sequential phases of the study, beginning with experiments on the robotic fish, followed by simulations on the pitching foil, and culminating in mathematical modeling.

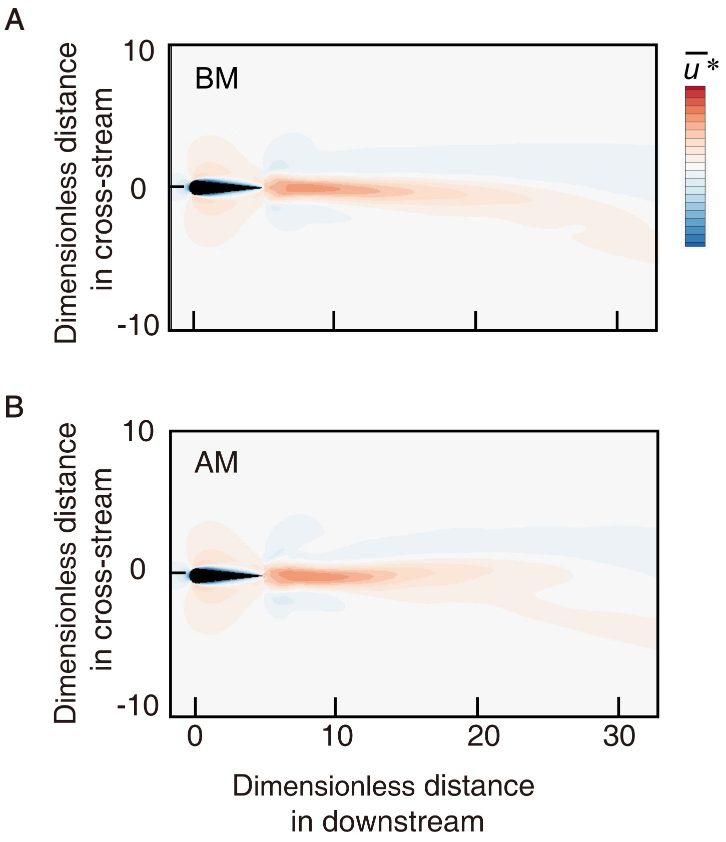

Fig. S2. Contours of the time-averaged relative streamwise velocity $\bar{u}^* = (\bar{u} - U)/U$, where $\bar{u}$ is the local time-averaged streamwise velocity. PM and AM with $(\tilde{f}, \tilde{A}, Re) = (10, 0.04, 10^3)$.

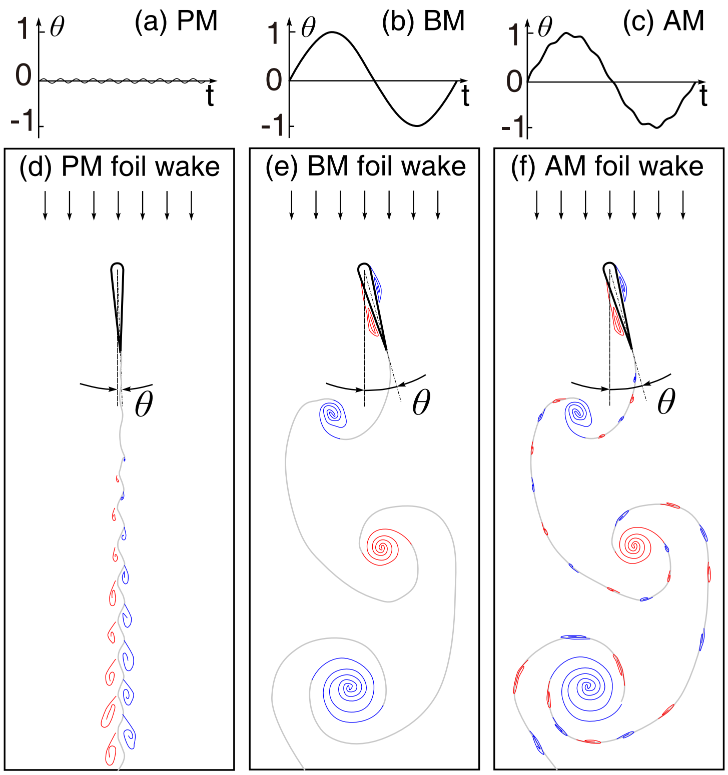

Fig. S3. (a-c) Sketch of kinematic scenarios under perturbation mode (PM), basic mode (BM), and accumulated mode (AM), respectively. (d-f) Schematic of wakes under different modes.

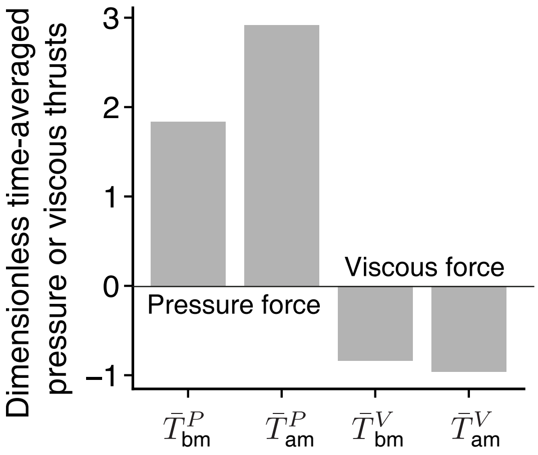

Fig. S4. Dimensionless time-averaged pressure-induced thrust and viscous drag in BM and AM $(\tilde{f}, \tilde{A}, Re) = (10, 0.04, 10^3)$, where quantities are dimensionalized by the time-mean thrust of the basic mode. For BM, the dimensionalized time-averaged pressure-induced thrust and viscous drag are 1.837 and -0.837, respectively, while the AM generates the dimensionless time-averaged pressure-induced thrust of 2.917 and viscous drag of -0.959.

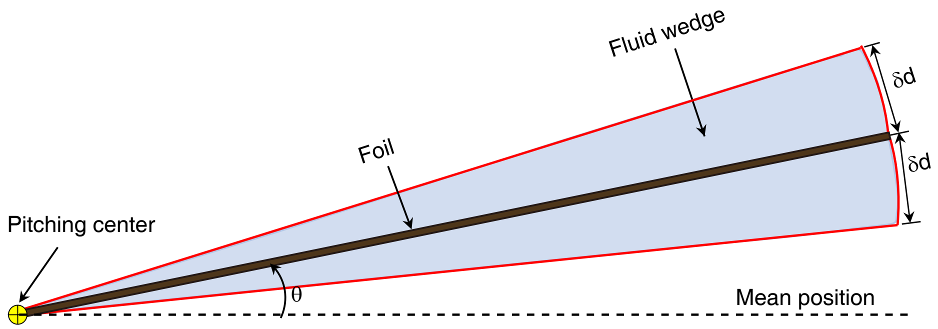

Fig. S5. Fluid wedge assumption where fluid moving with the foil with negligible thickness is considered as a fluid wedge of thickness of $2\delta d$.

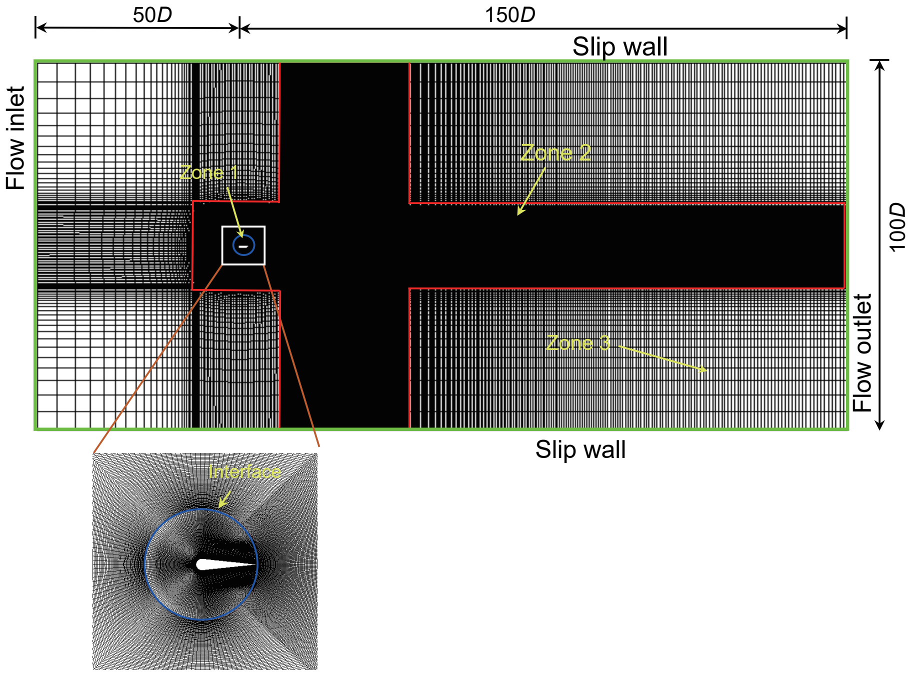

Fig. S6. Computational domain

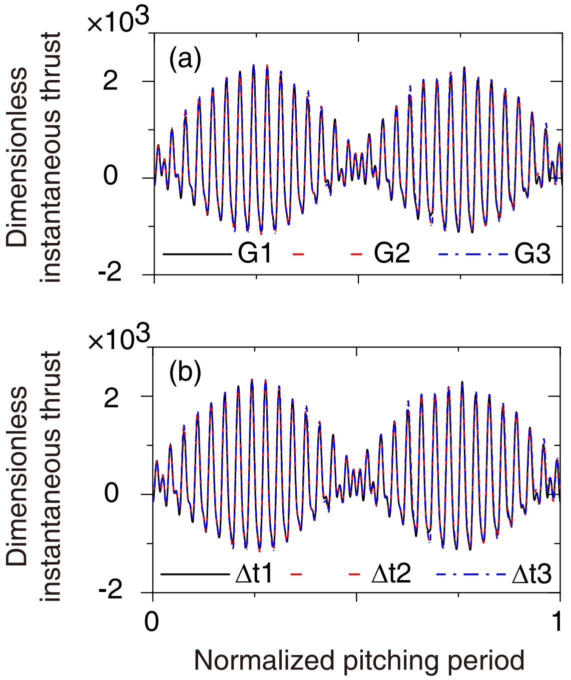

Fig. S7. (a) Grid independence and (b) time-step independence tests.

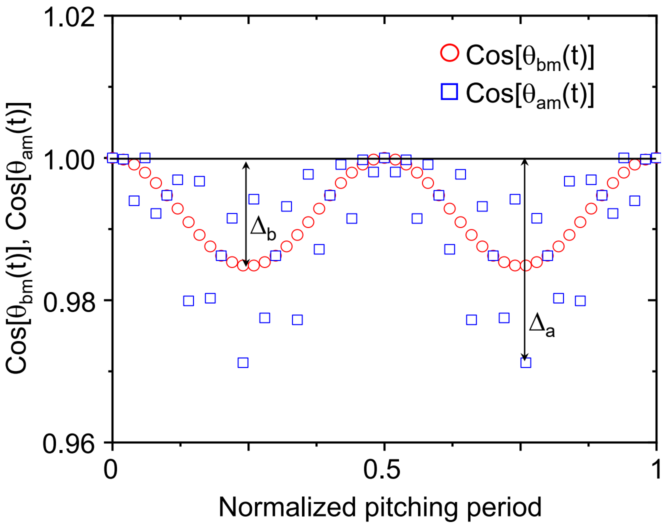

Fig. S8. Varying of $\cos[\theta_{bm}(t)]$ and $\cos[\theta_{am}(t)]$, where the maximal fluctuations of the $\cos[\theta_{bm}(t)]$ and $\cos[\theta_{am}(t)]$ are $\delta_b = 0.016$ and $\delta_a = 0.030$, respectively.

Table S1. Grid independent test.

Grid system	Dimensionalized time-averaged thrust coefficient $\times 10^2$	time-averaged thrust difference $(\%)$
G1	2.9599	7.11
G2	3.1657	0.65
G3	3.1864	–

Table S2. Time-step independent test.

Grid system	Dimensionalized time-averaged thrust coefficient $\times 10^2$	time-averaged thrust difference $(\%)$
$\delta t1$	3.1851	1.27
$\delta t2$	3.1657	0.53
$\delta t3$	3.1491	–

Movie S1. Basic Mode (BM) *vs* Accumulated Mode (AM) in experiments.

Movie S2. Basic Mode (BM) *vs* Accumulated Mode (AM) in simulations.
